# Supplementary figures and images for: Beyond the ‘big four’: Venom profiling of the medically important yet neglected Indian snakes reveals disturbing antivenom deficiencies
Source: PLoS Negl Trop Dis. 2019 Dec 5;13(12):e0007899. doi: 10.1371/journal.pntd.0007899 (PMC6894822; doi:10.1371/journal.pntd.0007899)

**S1. Fig**

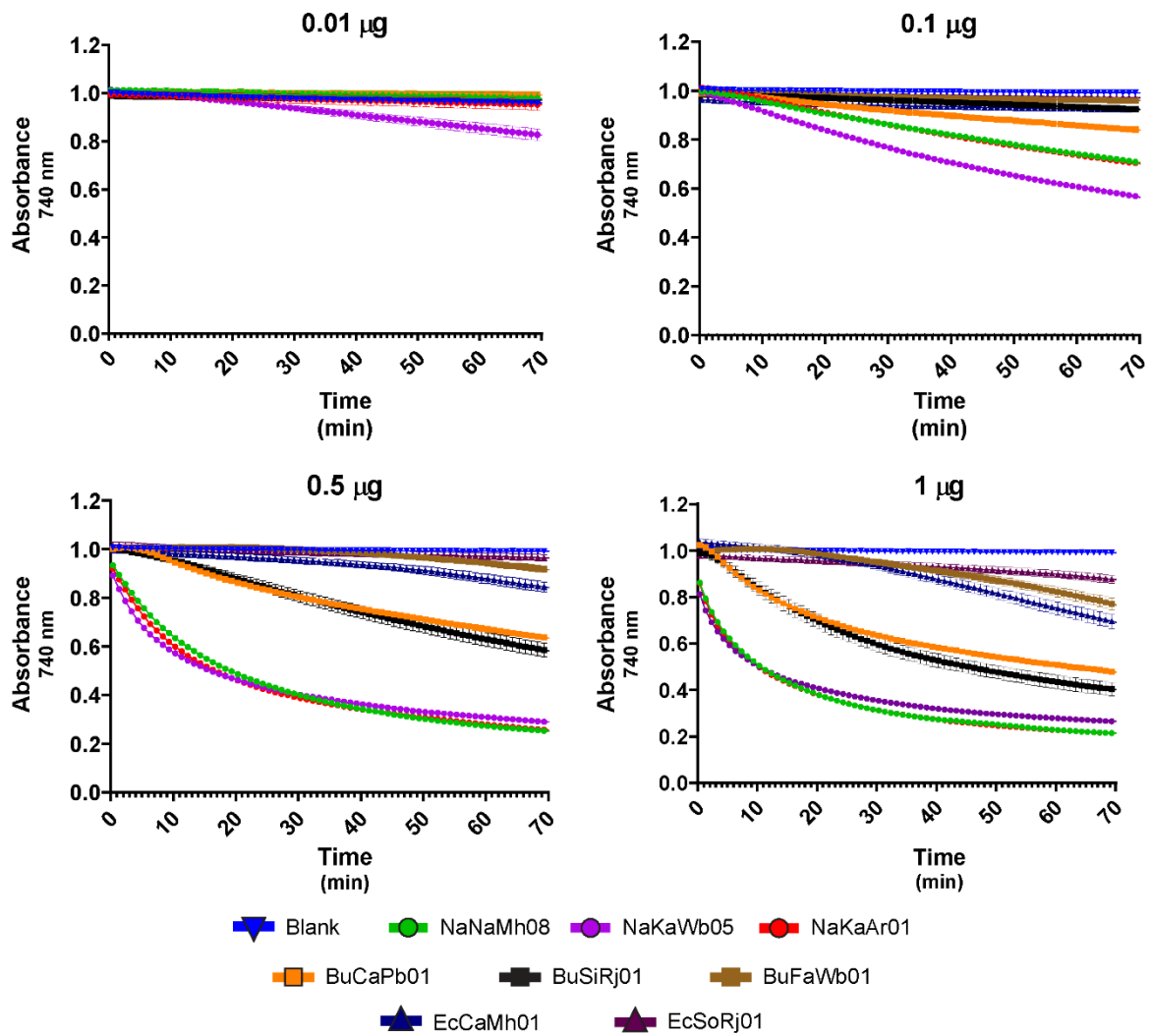

Supplement: S1 Fig — Kinetic activities of venom PLA2s of the medically important Naja spp., Bungarus spp. and Echis subspecies at 0.01 μg, 0.1 μg, 0.5 μg and 1 μg of venom concentrations are shown here. The change in the activity of venom PLA2s with time is measured by plotting the absorbance (OD) at 740 nm of the phospholipid substrate every minute over a time interval of 1 hour. The assay was performed in triplicates and the standard deviation is indicated by the error bars. (PDF) [file pntd.0007899.s001.pdf]

S2A-E. Fig

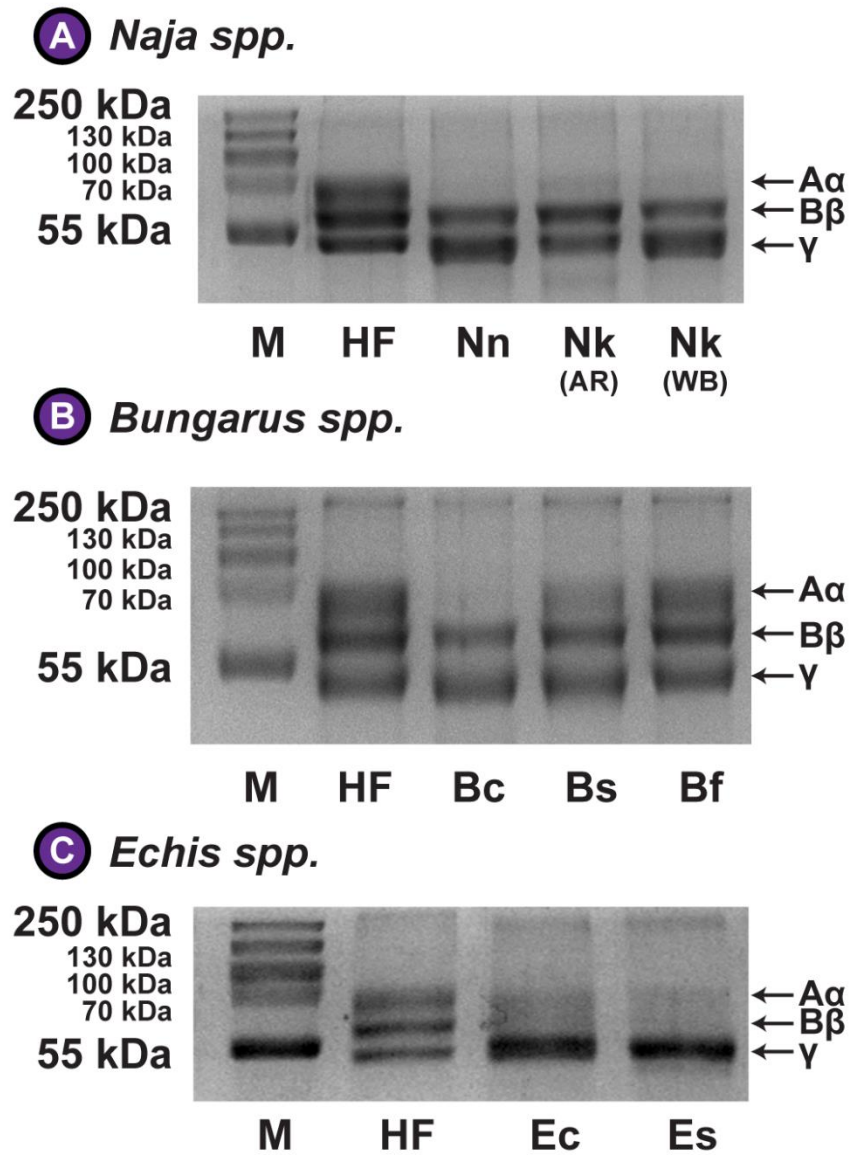

**D** *Echis carinatus*

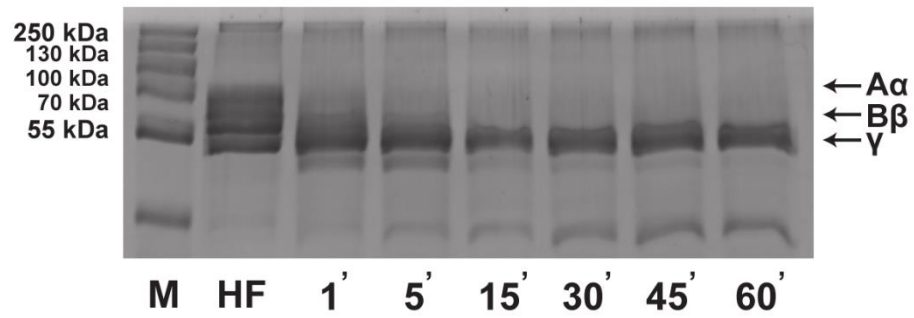

**E** *Echis carinatus sochureki*

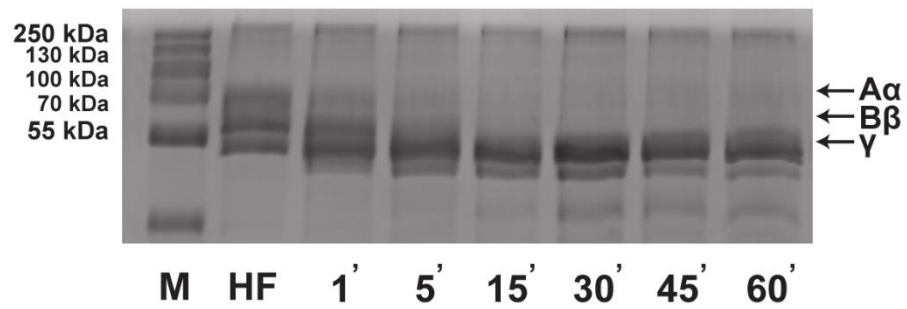

Supplement: S2 Fig — This figure shows fibrinogenolytic activities of (A) Naja spp., (B) Bungarus spp., and (C) Echis subspecies, and the time-dependent fibrinogenolytic activities of (D) E. carinatus and (E) E. c. sochureki venoms. M: Pre-stained protein ladder; HF: human fibrinogen; Nn: N. naja; Nk: N. kaouthia (AR); N. kaouthia (WB); Bc: B. caeruleus; Bs: B. sindanus; Bf: B. fasciatus; Ec: E. carinatus; Es: E. c. sochureki venoms; 1’ - 60’: Time lapsed in minutes. (PDF) [file pntd.0007899.s002.pdf]

**S3A. Fig**

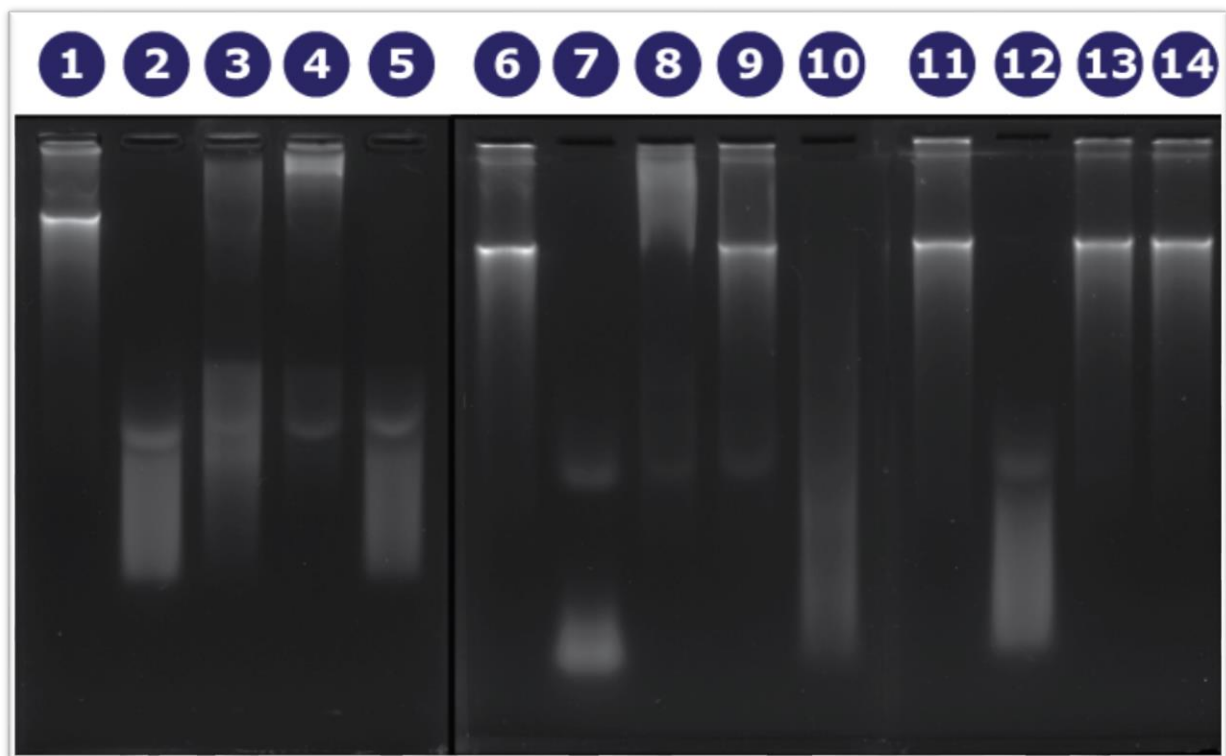

S3B. Fig

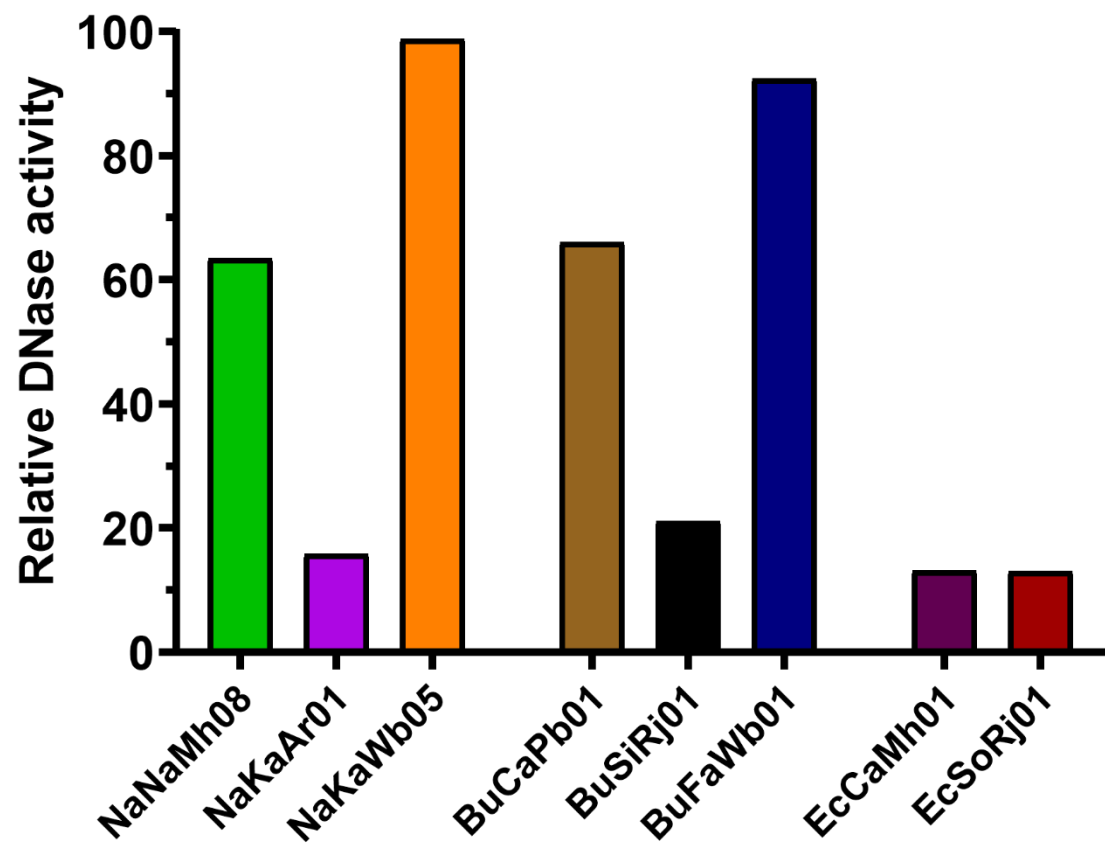

Supplement: S3 Fig — (A) Horizontal electrophoresis showing DNase activities. Lane 1: DNA (negative control); 2: DNA + 15 U DNase (positive control); 3: DNA + 50 μg/mL of NaNaMH08; 4: DNA + 50 μg/mL of NaKaAR01; 5: DNA + 50 μg/mL of NaKaWB05; 6: DNA (negative control); 7: DNA + 15 U DNase (positive control); 8: DNA + 50 μg/mL of BuCaPB01; 9: DNA + 50 μg/mL of BuSiRJ01; 10: DNA + 50 μg/mL of BuFaWB01; 11: DNA (negative control); 12: DNA + 15 U DNase (positive control); 13: DNA + 50 μg/mL of EcCaMH01; 14: DNA + 50 μg/mL of EcSoRJ01; (B) Relative DNase activities of Naja, Bungarus, and Echis venoms. (PDF) [file pntd.0007899.s003.pdf]

S4. Fig

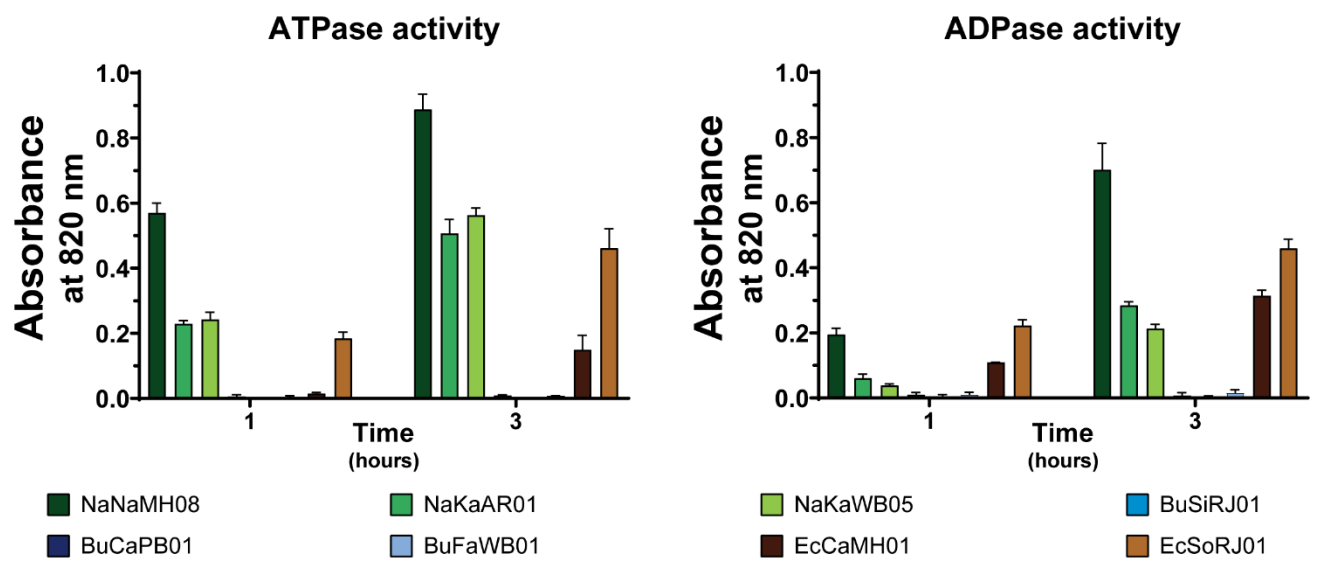

Supplement: S4 Fig — Assays were performed in triplicates, and the absorbance was measured at 820 nm after stopping the reactions at 1 and 3 hours. The standard deviation is indicated by the error bars. (PDF) [file pntd.0007899.s004.pdf]

S5. Fig

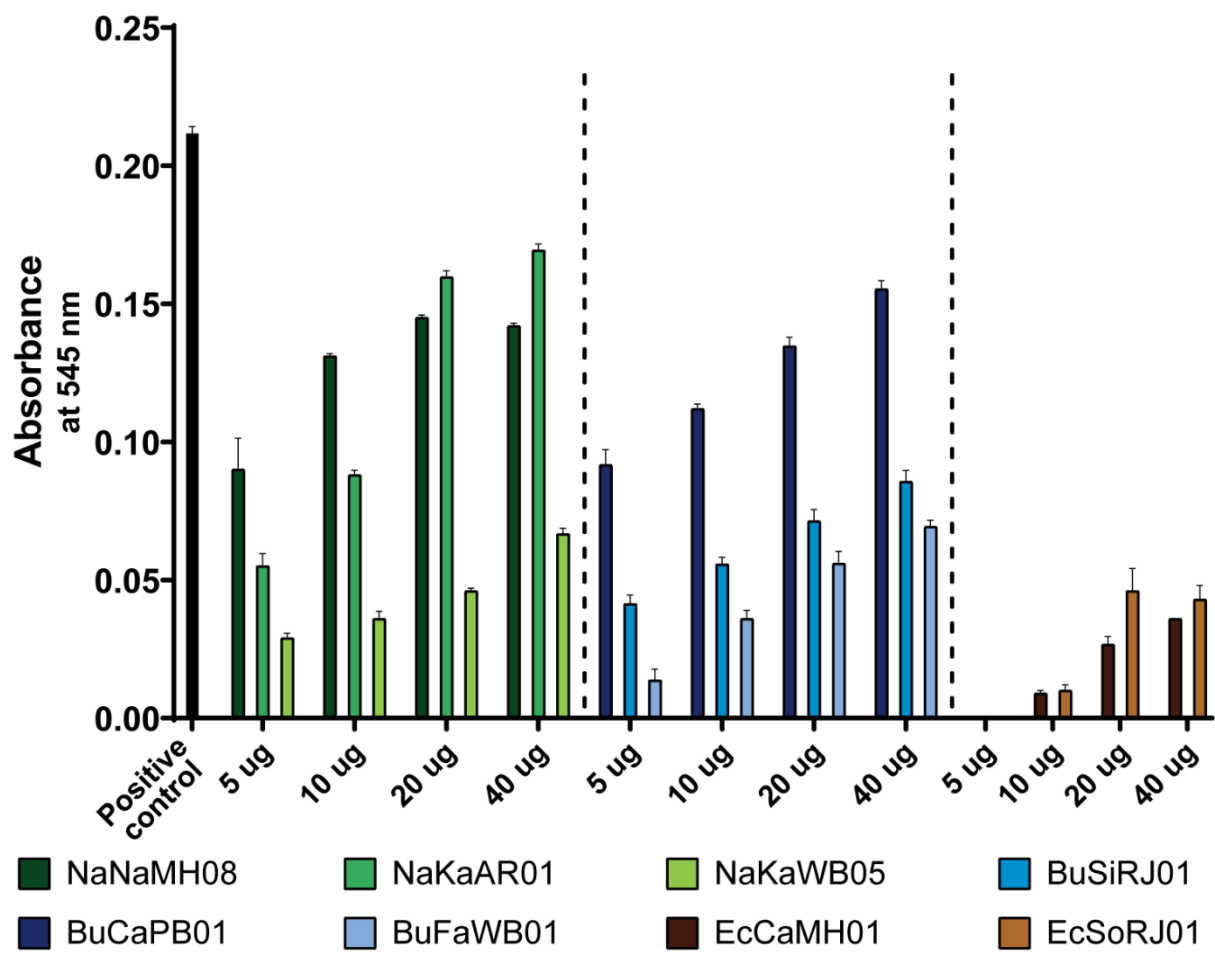

Supplement: S5 Fig — This figure depicts dose-dependent haemolytic activities of the venoms under study against 1% human erythrocyte solution. The activity was estimated based on absorbance at 545 nm. The assays were performed in triplicates and the standard deviation is indicated by the error bars. (PDF) [file pntd.0007899.s005.pdf]
