# Supplementary material for: Beyond the ‘big four’: Venom profiling of the medically important yet neglected Indian snakes reveals disturbing antivenom deficiencies
Source: PLoS Negl Trop Dis. 2019 Dec 5;13(12):e0007899. doi: 10.1371/journal.pntd.0007899 (PMC6894822; doi:10.1371/journal.pntd.0007899)

S6. Fig

Day 1: Severe ecchymosis and swelling

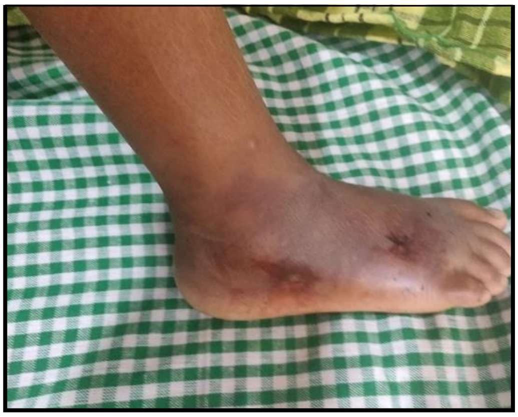

Day 2: Formation of blisters

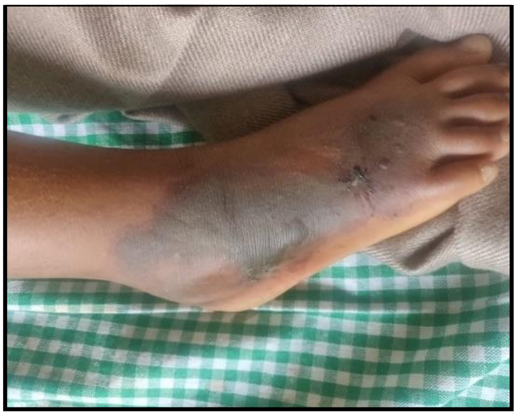

Day 3: Onset of necrosis

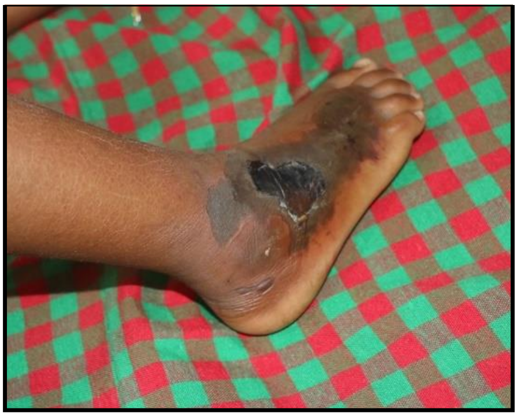

Day 4/5: Necrosis

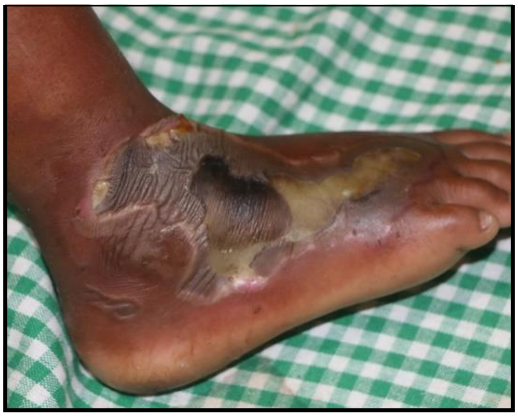

Supplement: S6 Fig — Pictures below of a four-year-old boy, bitten at the lateral side of the right foot, depict the cytotoxic effects associated with N. naja envenomations in West India (Maharashtra State). Despite the timely administration of antivenom, local necrosis was observed four to five days post envenomation, highlighting the inefficacy of commercial antivenoms in neutralizing cytotoxic symptoms caused by this Naja population. Interestingly, the proteomic characterization in this study revealed that only 5.4% of venom from this population of N. naja was found to comprise of cytotoxic 3FTxs. Photo and information credits: Dr. Sadananda Raut, Dr. Minoo Mehata memorial hospital, Narayangaon, Pune, Maharashtra. (PDF) [file pntd.0007899.s006.pdf]
