# Supplementary material for: Beyond the ‘big four’: Venom profiling of the medically important yet neglected Indian snakes reveals disturbing antivenom deficiencies
Source: PLoS Negl Trop Dis. 2019 Dec 5;13(12):e0007899. doi: 10.1371/journal.pntd.0007899 (PMC6894822; doi:10.1371/journal.pntd.0007899)
Supplement: S1 Table — A-B. Details of the snake venom and anti-snake venom tested. (PDF) [file pntd.0007899.s007.pdf]

S1A. Table

Species	Sample ID	Region	No. of individuals	Protein Content mg/mL
N. naja	NaNaMH08	Maharashtra (West India)	5	0.173
N. kaouthia	NaKaAR01	Arunachal Pradesh (Northeast India)	3	0.123
N. kaouthia	NaKaWB05	West Bengal (East India)	3	0.118
B. caeruleus	BuCaPB01	Punjab (North India)	4	0.166
B. sindanus	BuSiRJ01	Rajasthan (Northwest India)	2	0.274
B. fasciatus	BuFaWB01	West Bengal (East India)	1	0.187
E. carinatus	EcCaMH01	Maharashtra (West India)	17	0.82
E. c. sochureki	EcSoRJ01	Rajasthan (Northwest India)	10	0.93

S1B. Table

Manufacturer	Batch	Manufacture (M) and expiry (E) dates	Protein content	Marketed neutralizing efficacy	
			mg/mL	mg/mL	
**Bharat Serums and Vaccines Ltd.**	#A05316108	**M:** 07/2016<br>**E:** 06/2020	6.71 ± 0.24	*N. naja*	0.6 mg
**Haffkine Bio-Pharmaceutical Corporation Ltd.**	#AS180611	**M:** 06/2018<br>**E:** 11/2022	8.14 ± 0.09	*B. caeruleus*	0.45 mg
**Premium Serums & Vaccines Pvt. Ltd.**	#212013	**M:** 07/2016<br>**E:** 07/2020	8.16 ± 0.15	*D. russelii*	0.6 mg
**VINS Bioproducts Ltd.**	#01AS16046	**M:** 09/2016<br>**E:** 08/2020	9.69 ± 0.41	*E. carinatus*	0.45 mg
