# Supplementary material for: Beyond the ‘big four’: Venom profiling of the medically important yet neglected Indian snakes reveals disturbing antivenom deficiencies
Source: PLoS Negl Trop Dis. 2019 Dec 5;13(12):e0007899. doi: 10.1371/journal.pntd.0007899 (PMC6894822; doi:10.1371/journal.pntd.0007899)
Supplement: S2 Table — A-H. Components identified via tandem mass spectrometry of medically important snake venoms. For the identification of toxin classes present in the venom, PEAKS Studio X was used to search the raw MS/MS spectra against Uniprot’s SwissProt database (December 2018; www.uniprot.org), and the key statistics of these searches have been shown below. These include, identified protein groups, number of high-confidence peptides and unique peptides (mapping to only one group) supporting these protein groups, percentage of the protein sequence covered by supporting peptides (coverage), the area under the curve of the peptide feature found at the same m/z and retention time as the MS/MS scan (area), and the average molecular mass in KDa. Accession number, the name of species, and the toxin family of the matching uniport entry have also been provided. The percentage, indicated adjacent to the toxin family, corresponds to its proportion in the venoms of (A) N. naja from Maharashtra; (B) N. kaouthia from Arunachal Pradesh; (C) N. kaouthia from West Bengal; (D) E. carinatus from Maharashtra; (E) E. c. sochureki from Rajasthan; (F) B. caeruleus from Punjab; (G) B. sindanus from Rajasthan; (H) B. fasciatus from West Bengal, as determined by tandem mass spectrometry. (PDF) [file pntd.0007899.s008.pdf]

S2A-H Table

S2A. Venom composition of *N. Naja*

Sr.no.	Accession	Species	#Peptides	#Unique	Coverage (%)	Area	Avg. Mass (KDa)	Toxin type
**Cobra venom factor (CVF): 17.05%**								
1	Q91132	*Naja kaouthia*	46	29	31	1500000	184.517	Cobra venom factor
2	I2C090	*Ophiophagus hannah*	14	2	10	34000	183.927	Cobra venom factor
**Snake venom metalloproteinase (SVMP): 5.56%**								
3	F8RKW0	*Drysdalia coronoides*	2	1	3	4370	68.427	SVMP
4	D5LMJ3	*Naja atra*	5	3	8	63700	68.254	SVMP
5	F8RKW1	*Drysdalia coronoides*	2	1	3	4370	68.24	SVMP
6	Q10749	*Naja mossambica*	5	2	7	38600	68.176	SVMP
7	F8RKV9	*Drysdalia coronoides*	2	1	3	4370	68.087	SVMP
8	Q9PVK7	*Naja kaouthia*	17	12	28	261000	67.662	SVMP
9	D3TTC1	*Naja atra*	9	1	16	14300	66.292	SVMP
10	P82942	*Naja kaouthia*	11	4	25	110000	44.493	SVMP
**5'-nucleotidase: 2.37%**								
11	B6EWW8	*Gloydius brevicaudus*	11	11	20	213000	64.434	5'-nucleotidase
**L-amino-acid oxidase (LAAO): 3.44%**								
12	A8QL58	*Naja atra*	13	10	24	278000	51.439	L-amino-acid oxidase

13	P0DI91	*Naja oxiana*	5	2	47	31400	11.216	L-amino-acid oxidase
**Snake venom serine protease (SVSP): 0.58%**								
14	A8QL53	*Naja atra*	2	1	11	5050	31.137	SVSP
15	A8QL57	*Bungarus multicinctus*	2	1	11	5050	31.01	SVSP
16	A8QL56	*Ophiophagus hannah*	2	1	6	7730	28.656	α- and β-fibrinogenase
17	Q09GK1	*Philodryas olfersii*	2	1	6	7730	28.447	SVSP
18	P00761	*Sus scrofa*	2	2	13	26500	24.409	Trypsin
**Cysteine-rich secretory protein (CRISP): 2.39%**								
19	Q7T1K6	*Naja atra*	9	5	42	72700	26.882	CRISP
20	Q7ZT98	*Ophiophagus hannah*	5	1	15	23200	26.869	CRISP
21	P84805	*Naja kaouthia*	9	5	42	72700	26.846	CRISP
22	P84808	*Naja kaouthia*	6	6	39	46500	26.216	CRISP
**Phospholipase A2 (PLA2): 14.54%**								
23	Q3C2C2	*Acanthaster planci*	1	1	4	70900	17.83	PLA2
24	P59172	*Echis leakeyi*	1	1	5	70900	15.945	Acidic PLA2
25	P59170	*Echis sochureki*	1	1	5	70900	15.895	Acidic PLA2
26	Q90ZZ9	*Echis coloratus*	1	1	5	70900	15.77	PLA2
27	P0CAR9	*Cerastes cerastes*	1	1	5	70900	15.365	Acidic PLA2
28	Q9YGJ7	*Daboia palaestinae*	1	1	5	70900	15.35	Acidic PLA2

29	F8QN53	*Vipera renardi*	1	1	5	70900	15.316	Acidic PLA2
30	P21789	*Cerastes cerastes*	1	1	6	70900	13.534	Acidic PLA2
31	P24293	*Eristicophis macmahoni*	1	1	6	70900	13.533	Acidic PLA2
32	Q5G290	*Naja sagittifera*	8	1	27	14300	13.501	Acidic PLA2
33	P15445	*Naja naja*	17	6	64	629000	13.346	Acidic PLA2
34	P25498	*Naja oxiana*	6	2	34	26900	13.229	Acidic PLA2
**Natriuretic peptide: 0.07%**								
35	D9IX97	*Naja atra*	1	1	5	6550	17.345	Natriuretic peptide
**Vespryn: 0.60%**								
36	P82885	*Naja kaouthia*	2	2	29	53600	12.038	Vespryn
**Three-finer toxins (3FTx): 47.21%**								
37	Q9W717	*Naja atra*	2	2	38	32400	9.695	Short-chain α-neurotoxin
38	Q9W716	*Naja atra*	5	2	46	112000	9.333	Cytotoxin
39	P62375	*Naja atra*	5	2	46	112000	9.323	Cytotoxin
40	Q91996	*Naja atra*	5	2	46	112000	9.305	Cardiotoxin
41	P25672	*Naja haje*	10	1	75	176000	7.889	Long-chain α-neurotoxin
42	P25668	*Naja haje*	13	1	80	70000	7.847	Long-chain α-neurotoxin
43	P01391	*Naja kaouthia*	13	1	79	923000	7.831	Long-chain α-neurotoxin
44	P25674	*Naja haje*	6	1	51	9620	7.821	Long-chain α-neurotoxin
45	P25669	*Naja haje*	13	1	80	741000	7.821	Long-chain α-neurotoxin

46	P34074	*Naja annulata*	6	1	51	9620	7.768	Long-chain α-neurotoxin
47	P29181	*Naja naja*	3	1	35	1950	7.637	Long-chain α-neurotoxin
48	P82464	*Naja kaouthia*	5	5	71	47300	7.624	Muscarinic neurotoxin
49	P82463	*Naja kaouthia*	2	2	42	219000	7.298	Muscarinic neurotoxin
50	P62377	*Naja naja*	5	2	61	112000	7.014	Cytotoxin
51	P01427	*Naja oxiana*	5	5	61	1530000	6.885	Short-chain α-neurotoxin
52	P62390	*Naja annulifera*	4	2	47	10700	6.842	Cytotoxin
53	P62394	*Naja haje*	4	2	47	10700	6.842	Cytotoxin
54	P86538	*Naja naja*	12	2	80	18900	6.711	Cytotoxin
**Kunitz-type serine protease inhibitor: 6.20%**								
55	P19859	Naja naja	3	2	51	31100	6.508	Kunitz
56	P20229	Naja naja	8	7	84	527000	6.371	Kunitz

S2B. Venom composition of *N. kaouthia* (Arunachal Pradesh)

Sr.no.	Accession	Species	#Peptides	#Unique	Coverage (%)	Area	Avg. mass (KDa)	Toxin type
**Cobra venom factor (CVF): 7.90%**								
1	Q01833	*Naja naja*	16	1	12	8600	184.926	Cobra venom factor
2	Q0ZZJ6	*Austrelaps superbus*	10	1	5	16300	184.725	Cobra venom factor
3	Q91132	*Naja kaouthia*	62	39	49	885000	184.517	Cobra venom factor
4	I2C090	*Ophiophagus hannah*	16	2	11	40700	183.927	Cobra venom factor
**Venom phosphodiesterase (PDE): 0.43%**								
5	J3SEZ3	*Crotalus adamanteus*	2	2	3	25800	96.373	Phosphodiesterase
6	J3SBP3	*Crotalus adamanteus*	2	2	3	25800	91.752	Phosphodiesterase
**Snake venom metalloproteinase (SVMP): 4.87%**								
7	B8K1W0	*Daboia russelii*	2	1	2	29000	69.555	SVMP
8	D3TTC2	*Naja atra*	14	2	25	36900	69.181	SVMP
9	D5LMJ3	*Naja atra*	4	4	3	45200	68.254	SVMP
10	Q10749	*Naja mossambica*	4	2	6	107000	68.176	SVMP
11	Q9DGB9	*Crotalus atrox*	1	1	3	4040	67.96	SVMP
12	F8S108	*Crotalus adamanteus*	1	1	3	4040	67.91	SVMP
13	Q9PVK7	*Naja kaouthia*	26	12	37	280000	67.662	SVMP
14	D3TTC1	*Naja atra*	4	1	10	9400	66.292	SVMP

15	D6PXE8	*Naja atra*	4	1	10	9400	66.246	SVMP
16	Q076D1	*Crotalus terrificus*	1	1	4	4040	46.565	SVMP
17	C5H5D1	*Crotalus cascavella*	1	1	4	4040	46.165	SVMP
18	P82942	*Naja kaouthia*	7	4	31	53300	44.493	SVMP
**Acetylcholinesterase (AChE): 0.03%**								
19	Q92035	*Bungarus fasciatus*	2	2	6	3860	68.074	Acetylcholinesterase
**5'-nucleotidase: 1.17%**								
20	B6EWW8	*Gloydius brevicaudus*	11	11	19	135000	64.434	5'-nucleotidase
21	Q05927	*Bos taurus*	1	1	4	6120	62.966	5'-nucleotidase
**L-amino-acid oxidase (LAAO): 0.55%**								
22	A8QL58	*Naja atra*	7	6	24	57600	51.439	L-amino-acid oxidase
23	P0DI91	*Naja oxiana*	2	1	33	8510	11.216	L-amino-acid oxidase
**Nerve growth factor (NGF): 2.25%**								
24	Q5YF89	*Naja sputatrix*	9	1	38	8400	27.03	Nerve growth factor
25	P61898	*Naja atra*	10	2	76	131000	13.064	Nerve growth factor
26	P61899	*Naja kaouthia*	10	2	76	131000	13.064	Nerve growth factor
**Cysteine-rich secretory proteins (CRISP): 13.12%**								
27	Q7T1K6	*Naja atra*	25	25	87	1030000	26.882	CRISP
28	P84808	*Naja kaouthia*	12	12	72	549000	26.216	CRISP

**Phospholipase A2 (PLA2): 0.81%**								
29	P00596	*Naja kaouthia*	23	1	62	9500	16.271	Acidic PLA2
30	Q9I900	*Naja sputatrix*	21	1	52	21700	16.097	Acidic PLA2
31	P00597	*Naja kaouthia*	34	2	79	21500	16.016	Acidic PLA2
32	P00598	*Naja atra*	34	2	79	21500	16.013	Acidic PLA2
33	P15445	*Naja naja*	26	1	84	14400	13.346	Acidic PLA2
34	P25498	*Naja oxiana*	15	1	50	9060	13.229	Acidic PLA2
**Vespryn: 1.30%**								
35	P82885	*Naja kaouthia*	5	5	56	156000	12.038	Vespryn
**Three-finger toxins (3FTx): 67.56%**								
36	Q9DEQ3	*Naja atra*	4	3	59	22000	9.962	Short-chain α-neurotoxin
37	Q9W727	*Bungarus multicinctus*	4	3	59	22000	9.934	Muscarinic neurotoxin
38	Q9YGI2	*Naja atra*	5	2	33	6480	9.845	Long-chain α-neurotoxin
39	O93422	*Naja atra*	5	2	33	6480	9.815	Long-chain α-neurotoxin
40	Q9PTT0	*Naja naja*	5	1	46	510000	9.262	Short-chain α-neurotoxin
41	P60770	*Naja atra*	5	1	46	510000	9.262	Short-chain α-neurotoxin
42	P60771	*Naja kaouthia*	5	1	46	510000	9.262	Short-chain α-neurotoxin
43	P49122	*Naja atra*	5	2	32	56800	9.086	Cytotoxin
44	P80245	*Naja atra*	18	1	53	19100	8.98	Cytotoxin

45	P25668	*Naja naja*	3	2	46	11900	7.847	Long-chain α-neurotoxin
46	P01391	*Naja kaouthia*	12	5	76	878000	7.831	Long-chain α-neurotoxin
47	P25674	*Naja haje*	5	1	51	14200	7.821	Long-chain α-neurotoxin
48	P34074	*Naja annulata*	5	1	51	14200	7.768	Long-chain α-neurotoxin
49	P29181	*Naja naja*	1	1	14	8570	7.637	Long-chain α-neurotoxin
50	P82464	*Naja kaouthia*	3	2	72	24200	7.624	Muscarinic neurotoxin
51	P25679	*Naja kaouthia*	7	7	28	2320000	7.438	Long-chain α-neurotoxin
52	P01400	*Naja melanoleuca*	4	1	43	25100	7.43	Long-chain α-neurotoxin
53	P82462	*Naja kaouthia*	8	6	74	531000	7.366	Muscarinic neurotoxin
54	P82463	*Naja kaouthia*	8	8	82	355000	7.298	Muscarinic neurotoxin
55	P14541	*Naja kaouthia*	5	2	37	10400	6.994	Cytotoxin
56	Q9PSN6	*Naja sputatrix*	5	1	61	67400	6.958	Short-chain α-neurotoxin
57	P59276	*Naja kaouthia*	6	1	61	7340	6.859	Short-chain α-neurotoxin
58	P01452	*Naja mossambica*	6	2	38	98200	6.715	Cytotoxin
59	P86538	*Naja naja*	14	8	63	2100000	6.711	Cytotoxin

S2C. Venom composition of *N. kaouthia* (West Bengal)

Sr.no.	Accession	Species	#Peptides	#Unique	Coverage (%)	Area	Avg. mass (KDa)	Toxin type
**Cobra venom factor (CVF): 3.16%**								
1	J3S836	*Crotalus adamanteus*	10	1	6	1890	184.922	Venom factor
2	Q91132	*Naja kaouthia*	52	32	38	562000	184.517	Cobra venom factor
3	I2C090	*Ophiophagus hannah*	14	2	11	31500	183.927	Cobra venom factor
**Snake venom metalloproteinase (SVMP): 1.31%**								
4	D5LMJ3	*Naja atra*	5	4	11	17300	68.254	SVMP
5	F8RKW1	*Drysdalia coronoides*	2	1	4	1090	68.24	SVMP
6	Q9PVK7	*Naja kaouthia*	15	14	29	200000	67.662	SVMP
7	D3TTC1	*Naja atra*	2	1	5	12200	66.292	SVMP
8	D6PXE8	*Naja atra*	2	1	5	12200	66.246	SVMP
9	P82942	*Naja kaouthia*	2	1	8	2810	44.493	SVMP
**5'-nucleotidase: 0.22%**								
10	B6EWW8	*Gloydius brevicaudus*	9	9	18	41000	64.434	5'-nucleotidase
**Phospholipase B (PLB): 0.02%**								
11	F8J2D3	*Drysdalia coronoides*	1	1	5	3510	64.103	Phospholipase B
**L-amino-acid oxidase (LAAO): 0.02%**								

12	A8QL58	*Naja atra*	2	2	10	3840	51.439	L-amino acid oxidase
**Cysteine-rich secretory proteins (CRISP): 1.20%**								
13	Q7T1K6	*Naja atra*	9	9	56	79400	26.882	CRISP
14	P84805	*Naja kaouthia*	9	9	56	79400	26.846	CRISP
15	Q7ZZN8	*Naja atra*	6	6	42	33200	26.246	CRISP
16	P84808	*Naja kaouthia*	6	6	42	33200	26.216	CRISP
**Snake venom serine protease (SVSP): 0.01%**								
17	P06871	*Canis familiaris*	1	1	1	891	26.170	Trypsin
18	P00761	*Sus scrofa*	1	1	4	891	24.409	Trypsin
**C-type lectin (CTL): 0.20%**								
19	Q90WI6	*Bungarus multicinctus*	1	1	5	7410	18.706	C-type lectin
20	D2YVI2	*Pseudechis australis*	1	1	5	7410	18.687	C-type lectin
21	Q90WI8	*Bungarus fasciatus*	1	1	5	7410	18.638	C-type lectin
22	D2YVJ6	*Pseudechis porphyriacus*	1	1	5	7410	18.617	C-type lectin
23	D2YVK1	*Hoplocephalus stephensii*	1	1	5	7410	18.567	C-type lectin
**Natriuretic peptide (NP): 0.04%**								
24	D9IX97	*Naja atra*	3	3	14	6770	17.345	Natriuretic peptide
**Phospholipase A2 (PLA2): 3.08%**								
25	P00596	*Naja kaouthia*	24	2	62	24500	16.271	Acidic PLA2
26	Q9I900	*Naja sputatrix*	22	2	58	555000	16.097	Acidic PLA2

**Nerve growth factor (NGF): 0.03%**								
27	P61898	*Naja atra*	1	1	9	3010	13.064	Nerve growth factor
28	P61899	*Naja kaouthia*	1	1	9	3010	13.064	Nerve growth factor
**Vespryn: 0.91%**								
29	P82885	*Naja kaouthia*	4	4	56	171000	12.038	Vespryn
**Three-finger toxins (3FTx): 89.72%**								
30	Q9W717	*Naja atra*	3	3	40	89200	9.695	Short-chain α-neurotoxin
31	F8J2E6	*Drysdalia coronoides*	3	1	11	239000	9.526	Long-chain α-neurotoxin
32	Q9PTT0	*Naja naja*	7	5	47	812000	9.262	Short-chain α-neurotoxin
33	P60770	*Naja atra*	7	5	47	812000	9.262	Short-chain α-neurotoxin
34	P60771	*Naja kaouthia*	7	5	47	812000	9.262	Short-chain α-neurotoxin
35	Q9PST4	*Naja sputatrix*	8	1	41	121000	9.054	Cytotoxin
36	Q9PST3	*Naja sputatrix*	8	1	41	121000	9.042	Cytotoxin
37	A0A0U5AUY6	*Naja naja*	8	1	46	121000	8.041	Cytotoxin
38	P25668	*Naja naja*	10	1	72	5850	7.847	Long-chain α-neurotoxin
39	P01391	*Naja kaouthia*	32	9	99	10500000	7.831	Long-chain α-neurotoxin
40	P25674	*Naja haje*	10	3	55	405000	7.821	Long-chain α-neurotoxin

41	P82464	*Naja kaouthia*	2	2	29	22700	7.624	Muscarinic neurotoxin
42	P01401	*Naja haje*	6	1	45	2550	7.546	Long-chain α-neurotoxin
43	P01400	*Naja melanoleuca*	7	2	62	74600	7.43	Long-chain α-neurotoxin
44	P82462	*Naja kaouthia*	4	2	63	304000	7.366	Muscarinic neurotoxin
45	P82463	*Naja kaouthia*	5	5	75	164000	7.298	Muscarinic neurotoxin
46	P01431	*Naja mossambica*	4	2	29	20000	7.081	Short-chain α-neurotoxin
47	P59276	*Naja kaouthia*	10	10	61	1530000	6.859	Short-chain α-neurotoxin
48	P86541	*Naja naja*	8	1	55	121000	6.764	Cytotoxin
49	P60308	*Naja atra*	3	1	27	17300	6.755	Cytotoxin
50	P86538	*Naja naja*	10	5	62	206000	6.711	Cytotoxin
**Kunitz-type serine protease inhibitor: 0.1%**								
51	Q5ZPJ7	*Naja atra*	2	2	59	18200	8.815	Kunitz

S2D. Venom composition of *Echis carinatus*

Sr.no.	Accession	Species	#Peptides	#Unique	Coverage (%)	Area	Avg. mass (KDa)	Toxin type
**Hyaluronidase (HYL): 3.91%**								
1	Q9UHN6	*Homo sapiens*	1	1	1	6510	154.373	Hyaluronidase
2	Q5FWI3	*Mus musculus*	1	1	1	6510	153.8	Hyaluronidase
3	A3KPQ7	*Danio rerio*	1	1	1	6510	152.327	Hyaluronidase
**Snake venom metalloproteinase (SVMP): 18.69%**								
4	Q90495	*Echis carinatus*	3	2	3	16100	69.463	SVMP
5	Q2UXQ5	*Echis ocellatus*	3	1	6	6710	69.426	SVMP
6	Q2UXR0	*Echis ocellatus*	5	1	7	1490	68.751	SVMP
7	Q4VM08	*Macrovipera lebetina*	5	4	6	5350	68.71	SVMP
8	Q10749	*Naja mossambica*	1	1	2	10300	68.176	SVMP
9	Q98UF9	*Bothrops jararaca*	4	2	6	19000	67.695	SVMP
10	Q9PVK7	*Naja kaouthia*	1	1	2	10300	67.662	SVMP
11	P0DM97	*Bitis arietans*	1	1	8	5150	58.718	SVMP
12	P86092	*Bothrops leucurus*	4	2	10	19000	36.262	SVMP
**L-amino-acid oxidase (LAAO): 25.13%**								
13	Q4JHE3	*Oxyuranus scutellatus*	1	1	2	25400	59.07	L-amino-acid oxidase
14	Q4JHE2	*Notechis scutatus*	1	1	2	25400	59.058	L-amino-acid oxidase
15	A8QL51	*Bungarus multicinctus*	1	1	2	25400	58.811	L-amino-acid oxidase
16	A8QL52	*Bungarus fasciatus*	1	1	2	25400	58.764	L-amino-acid oxidase

17	G8XQX1	*Daboia russelii*	5	5	9	17600	56.888	L-amino-acid oxidase
18	P0C2D6	*Protobothrops mucrosquamatus*	1	1	50	6370	2.931	L-amino-acid oxidase
**Snake venom serine (SVSP): 3.01%**								
19	Q6T6S7	*Bitis gabonica*	1	1	3	2290	28.982	SVSP
20	B5U6Y3	*Echis ocellatus*	1	1	3	3310	28.255	SVSP
21	Q8AY79	*Trimeresurus stejnegeri*	1	1	4	2520	28.028	β-fibrinogenase
22	P00761	*Sus scrofa*	1	1	3	3190	24.409	Trypsin
23	P86497	*Bitis rhinoceros*	1	1	16	3730	9.978	SVSP
**Snake lectins (Snaclec): 12.04%**								
24	Q7T2Q1	*Echis multisquamatus*	1	1	4	1320	18.214	Snaclec
25	Q6T7B6	*Bitis gabonica*	1	1	6	9820	18.157	Snaclec
26	B4XSZ0	*Macrovipera lebetina*	1	1	6	9820	17.759	Snaclec
27	B4XSY9	*Macrovipera lebetina*	1	1	6	9820	17.711	Snaclec
28	W5XCJ6	*Macrovipera lebetina*	1	1	6	9820	17.553	Snaclec
29	B0VXV2	*Sistrurus edwardsii*	1	1	10	2350	16.939	Snaclec
30	P81798	*Echis multisquamatus*	1	1	41	17200	3.458	Snaclec
**Phospholipase A2 (PLA2): 29.21%**								
31	Q7T3S7	*Echis carinatus*	8	8	46	146000	15.523	Acidic PLA2
**Disintegrins: 8.02%**								
32	Q5EE07	*Echis carinatus*	3	3	61	39000	7.127	Disintegrin
33	P0C6R5	*Echis multisquamatus*	2	2	19	1080	5.74	Disintegrin

S2E. Venom composition of *E. c. sochureki*

Sr.no.	Accession	Species	#Peptides	#Unique	Coverage (%)	Area	Avg. Mass (KDa)	Toxin type
**Hyaluronidase (HYL): 1.71%**								
1	Q9UHN6	*Homo sapiens*	1	1	1	5460	154.373	Hyaluronidase
2	Q5FWI3	*Mus musculus*	1	1	1	5460	153.8	Hyaluronidase
3	A3KPQ7	*Danio rerio*	1	1	1	5460	152.327	Hyaluronidase
**Snake venom metalloproteinase (SVMP): 2.96%**								
4	B8K1W0	*Daboia russelii*	1	1	2	24200	69.555	SVMP
5	Q90495	*Echis carinatus*	1	1	2	4110	69.463	SVMP
6	P0DM89	*Gloydius brevicaudus*	1	1	4	1970	46.785	SVMP
**L-amino-acid oxidase (LAAO): 12.65%**								
7	Q4JHE3	*Oxyuranus scutellatus*	1	1	2	26200	59.07	L-amino-acid oxidase
8	Q4JHE2	*Notechis scutatus*	1	1	2	26200	59.058	L-amino-acid oxidase
9	A8QL51	*Bungarus multicinctus*	1	1	2	26200	58.811	L-amino-acid oxidase
10	A8QL52	*Bungarus fasciatus*	1	1	2	26200	58.764	L-amino-acid oxidase
11	G8XQX1	*Daboia russelii*	7	3	15	15700	56.888	L-amino-acid oxidase
12	B5U6Y8	*Echis ocellatus*	5	1	11	373	56.523	L-amino-acid oxidase

**Snake venom serine protease (SVSP): 7.43%**								
13	O13061	*Trimeresurus gramineus*	1	1	3	4310	28.934	SVSP
14	P18965	*Daboia siamensis*	1	1	3	2740	28.823	Factor V activator
15	P0C5B4	*Gloydius shedaoensis*	1	1	3	2740	28.616	Thrombin-like
16	Q8JH85	*Macrovipera lebetina*	1	1	3	4310	28.608	α-fibrinogenase
17	Q8UVX1	*Gloydius ussuriensis*	1	1	3	2740	28.53	SVSP
18	E5L0E3	*Daboia siamensis*	1	1	3	4310	28.496	α-fibrinogenase
19	E0Y420	*Macrovipera lebetina*	1	1	3	4310	28.352	SVSP
20	E0Y419	*Macrovipera lebetina*	1	1	4	4310	28.297	β-fibrinogenase
21	B5U6Y3	*Echis ocellatus*	1	1	3	4310	28.255	SVSP
22	E5L0E4	*Daboia siamensis*	1	1	4	4310	28.035	β-fibrinogenase
23	P81176	*Gloydius blomhoffii*	1	1	12	11100	26.483	Thrombin-like
24	P18964	*Daboia siamensis*	1	1	3	2740	26.182	Factor V activator
25	P00761	*Sus scrofa*	1	1	3	2670	24.409	Trypsin
26	P86497	*Bitis rhinoceros*	2	2	28	16100	9.978	SVSP
**Cysteine-rich secretory proteins (CRISP): 6.24%**								
27	P0DMT4	*Echis coloratus*	6	6	30	59600	24.699	CRISP

**Snake lectin (Snaclec): 3.32%**								
28	Q6T7B6	*Bitis gabonica*	1	1	6	5280	18.157	Snaclec
29	B4XSZ1	*Macrovipera lebetina*	1	1	7	2120	17.778	Snaclec
30	B4XSZ0	*Macrovipera lebetina*	1	1	6	5280	17.759	Snaclec
31	B4XSY7	*Macrovipera lebetina*	1	1	7	2120	17.717	Snaclec
32	B4XSY9	*Macrovipera lebetina*	1	1	6	5280	17.711	Snaclec
33	W5XCJ6	*Macrovipera lebetina*	1	1	6	5280	17.553	Snaclec
34	B5U6Y7	*Echis ocellatus*	1	1	7	2120	17.507	Snaclec
35	B4XSZ9	*Macrovipera lebetina*	1	1	8	2120	15.643	Snaclec
36	B4XSY8	*Macrovipera lebetina*	1	1	8	2120	15.308	Snaclec
**C-type lectin (CTL): 0.22%**								
37	Q6X5T4	*Bitis arietans*	1	1	7	2120	17.232	C-type lectins
**Phospholipase A2 (PLA2): 61.87%**								
38	Q7T3S7	*Echis carinatus*	11	11	25	226000	15.523	Acidic PLA2
39	P48650	*Echis carinatus*	11	11	73	365000	13.819	Basic PLA2
**Disintegrin (DIS): 3.59%**								
40	Q6T6T3	*Bitis gabonica*	1	1	7	3000	13.792	Disintegrin
41	Q6T6T2	*Bitis gabonica*	1	1	7	3000	13.786	Disintegrin

42	P82465	*Echis sochureki*	1	1	8	3000	12.578	Disintegrin
43	P0C6A3	*Echis multisquamatus*	1	1	13	3000	7.469	Disintegrin
44	P81631	*Echis carinatus*	1	1	13	3000	7.424	Disintegrin
45	Q5EE07	*Echis carinatus*	1	1	14	3000	7.127	Disintegrin
46	P0C6A8	*Macrovipera obtusa*	1	1	14	3000	7.108	Disintegrin
47	P0C6A6	*Vipera berus*	1	1	14	3000	7.009	Disintegrin
48	P0C6R5	*Echis multisquamatus*	2	2	25	2070	5.74	Disintegrin
49	Q7LZK0	*Echis leakeyi*	2	2	27	2070	5.441	Disintegrin
50	P0C6R8	*Echis leakeyi*	2	2	27	2070	5.441	Disintegrin
51	P17347	*Echis sochureki*	2	2	27	2070	5.424	Disintegrin

S2F. Venom composition of *Bungarus caeruleus*

Sr.no.	Accession	Species	#Peptides	#Unique	Coverage (%)	Area	Avg. mass (KDa)	Toxin type
**Snake venom metalloproteinase (SVMP): 5.54%**								
1	A8QL49	*Bungarus multicinctus*	11	7	21	241000	68.99	SVMP
2	A8QL48	*Bungarus fasciatus*	5	1	10	2040	68.20	SVMP
**Acetylcholinesterase (AChE): 11.76%**								
3	Q92035	*Bungarus fasciatus*	19	19	36	516000	68.07	Acetylcholinesterase
**5'-nucleotidase: 1.00%**								
4	B6EWW8	*Gloydius brevicaudus*	9	9	19	44100	64.43	5'-nucleotidase
**L-amino-acid oxidase (LAAO): 4.86%**								
5	A8QL51	*Bungarus multicinctus*	31	11	47	184000	58.81	L-amino-acid oxidase
6	A8QL52	*Bungarus fasciatus*	20	1	35	29400	58.76	L-amino-acid oxidase
**Hyaluronidase (HYL): 0.71%**								
7	A3QVN6	*Echis leakeyi*	1	1	2	4450	52.69	Hyaluronidase
8	A3QVN3	*Cerastes cerastes*	1	1	2	4450	52.65	Hyaluronidase
9	A3QVN5	*Cerastes cerastes*	1	1	2	4450	52.62	Hyaluronidase
10	A3QVN4	*Cerastes cerastes*	1	1	2	4450	52.62	Hyaluronidase
11	A3QVN2	*Echis ocellatus*	1	1	2	4450	52.54	Hyaluronidase
12	A3QVN9	*Bitis arietans*	1	1	2	4450	52.31	Hyaluronidase
13	A3QVP0	*Bitis arietans*	1	1	2	4450	52.26	Hyaluronidase
**Venom nerve growth factor (NGF): 0.53%**								
14	P34128	*Bungarus multicinctus*	3	3	13	23200	27.51	Nerve growth factor

**Cysteine-rich secretory protein (CRISP): 0.43%**								
15	Q8AVA4	*Pseudechis australis*	4	1	12	3140	26.48	CRISP
16	Q8JI38	*Laticauda semifasciata*	3	1	23	2850	26.42	CRISP
17	Q2XXQ1	*Leioheterodon madagascariensis*	1	1	4	8260	24	CRISP
18	P81993	*Bungarus candidus*	8	2	55	4760	7.75	CRISP
**Vespryn: 1.59%**								
19	P83234	*Ophiophagus hannah*	2	1	16	28600	21.17	Vespryn
20	F8RKW2	*Drysdalia coronoides*	2	1	14	12500	21.12	Vespryn
21	P82885	*Naja kaouthia*	2	1	28	28600	12.04	Vespryn
**Phospholipase A2 (PLA2): 38.16%**								
22	Q9W7J3	*Pseudonaja textilis*	3	1	17	92000	17.14	Acidic PLA2
23	Q75S48	*Bungarus candidus*	4	1	24	17300	16.86	Acidic PLA2
24	Q8QFW4	*Bungarus caeruleus*	12	5	54	186000	16.36	Basic PLA2
25	Q7T2Q5	*Bungarus flaviceps*	1	1	10	14200	16.17	Acidic PLA2
26	Q8QFW3	*Bungarus caeruleus*	14	9	60	326000	16.12	Basic PLA2
27	Q8UW08	*Hydrophis hardwickii*	8	1	18	88700	16.11	Basic PLA2
28	Q8UW30	*Hydrophis hardwickii*	8	1	18	88700	16.08	Basic PLA2
29	Q802I1	*Bungarus candidus*	5	4	28	114000	15.64	Acidic PLA2
30	P00606	*Bungarus multicinctus*	13	1	38	82800	15.59	Acidic PLA2
31	Q6SLM2	*Bungarus caeruleus*	4	1	11	13200	15.06	Acidic PLA2
32	Q6SLM1	*Bungarus caeruleus*	11	3	36	536000	14.83	Basic PLA2
33	P00610	*Hydrophis schistosus*	8	1	23	88700	13.45	Basic PLA2
34	P14615	*Bungarus fasciatus*	3	2	21	20900	13.43	Neutral PLA2
35	P07037	*Aspidelaps scutatus*	5	1	16	6280	13.39	Acidic PLA2

**Three-finger toxins (3FTx): 32.56%**								
36	Q8JFX7	*Bungarus multicinctus*	1	1	9	5190	11.39	Muscarinic neurotoxin
37	Q9PW19	*Bungarus multicinctus*	1	1	9	5190	11.33	Cardiotoxin
38	P01384	*Notechis scutatus*	1	1	11	17700	10.29	Long-chain α-neurotoxin
39	Q9YGH9	*Bungarus multicinctus*	1	1	16	76600	9.87	Long-chain α-neurotoxin
40	Q9YGJ0	*Bungarus multicinctus*	1	1	16	76600	9.83	Long-chain γ-neurotoxin
41	O12963	*Bungarus multicinctus*	1	1	16	76600	9.76	Long-chain α-neurotoxin
42	A2CKF7	*Bungarus fasciatus*	1	1	10	388000	9.72	Long-chain α-neurotoxin
43	Q6IZ95	*Bungarus candidus*	1	1	10	388000	9.72	Long-chain α-neurotoxin
44	Q8AY56	*Bungarus candidus*	8	2	56	263000	9.58	Long-chain κ-neurotoxin
45	P01398	*Bungarus multicinctus*	7	2	56	2700	9.57	Long chain κ-neurotoxin
46	Q9YGI8	*Bungarus multicinctus*	4	4	35	82200	9.52	Short-chain α-neurotoxin
47	P15816	*Bungarus multicinctus*	4	1	48	11000	9.52	Long-chain κ-neurotoxin
48	D2N116	*Bungarus caeruleus*	1	1	17	6940	8.31	Long-chain α-neurotoxin
49	P01385	*Acanthophis antarcticus*	1	1	14	17700	8.14	Long-chain α-neurotoxin
50	P01383	*Naja melanoleuca*	1	1	14	11800	8.06	Long-chain α-neurotoxin

Kunitz-type serine protease inhibitor (Kunitz): 2.87%								
51	Q8AY43	*Bungarus candidus*	1	1	17	62900	9.08	Kunitz
52	Q8AY42	*Bungarus candidus*	1	1	17	62900	8.92	Kunitz

S2G. Venom composition of *B. sindanus*

Sr.no.	Accession	Species	#Peptides	#Unique	Coverage (%)	Area	Avg. mass (KDa)	Toxin type
**Snake venom metalloproteinase (SVMP): 1.91%**								
1	A8QL49	*Bungarus multicinctus*	14	14	30	275000	68.988	SVMP
**Acetylcholinesterase (AChE): 19.42%**								
2	Q92035	*Bungarus fasciatus*	44	44	55	2790000	68.074	Acetylcholinesterase
**5'-nucleotidase: 0.16%**								
3	B6EWW8	*Gloydius brevicaudus*	6	6	12	23100	64.434	5'-nucleotidase
**Phospholipase B (PLB): 0.16%**								
4	F8J2D3	*Drysdalia coronoides*	5	5	15	22400	64.103	Phospholipase B
**L-amino-acid oxidase (LAAO): 0.53%**								
5	A8QL52	*Bungarus fasciatus*	16	3	38	35800	58.764	L-amino acid oxidase
6	P0DI84	*Vipera ammodytes*	1	1	5	41000	54.748	L-amino acid oxidase
**Snake venom serine protease (SVSP): 3.62%**								
7	A8QL57	*Bungarus multicinctus*	4	4	13	15700	31.01	SVSP
8	P81782	*Bungarus candidus*	4	4	33	505000	7.285	SVSP

**Venom nerve growth factor (NGF): 0.74%**								
9	P34128	*Bungarus multicinctus*	3	3	13	106000	27.514	NGF
**Cysteine-rich secretory proteins (CRISP): 6.23%**								
10	Q8JI38	*Laticauda semifasciata*	13	5	35	84000	26.416	CRISP
11	P84808	*Naja kaouthia*	16	2	34	32000	26.216	CRISP
12	P81993	*Bungarus candidus*	10	2	42	219000	7.752	CRISP
**Vespryn: 3.07%**								
13	P83234	*Ophiophagus hannah*	4	3	16	159000	21.174	Vespryn
14	F8RKW2	*Drysdalia coronoides*	4	3	20	123000	21.119	Vespryn
15	P82885	*Naja kaouthia*	4	3	28	159000	12.038	Vespryn
**C-type lectin (CTL): 0.16%**								
16	Q90WI6	*Bungarus multicinctus*	4	4	35	23400	18.706	C-type lectin
**Phospholipase A2 (PLA2): 24.37%**								
17	Q75S48	*Bungarus candidus*	6	3	58	127000	16.855	Acidic PLA2
18	G9I930	*Micrurus tener*	1	1	6	7750	16.793	Basic PLA2
19	P00616	*Oxyuranus scutellatus*	1	1	8	29900	16.558	Acidic PLA2
20	Q8QFW3	*Bungarus caeruleus*	7	4	20	39300	16.12	Basic PLA2
21	P0C551	*Bungarus fasciatus*	8	4	44	41600	15.817	Acidic PLA2
22	Q802I1	*Bungarus candidus*	11	7	35	438000	15.641	Acidic PLA2

23	P00606	*Bungarus multicinctus*	4	1	37	34200	15.593	Acidic PLA2
24	Q8AY48	*Bungarus candidus*	11	5	31	1650000	15.257	Basic PLA2
25	Q90251	*Bungarus multicinctus*	11	4	45	393000	15.103	Acidic PLA2
26	Q6SLM1	*Bungarus caeruleus*	7	5	23	56000	14.827	Basic PLA2
27	P60043	*Naja sagittifera*	1	1	10	29900	14.003	Basic PLA2
28	P14615	*Bungarus fasciatus*	4	3	29	140000	13.425	Neutral PLA2
29	P20253	*Pseudechis australis*	2	1	25	454000	13.298	Basic PLA2
30	P00612	*Laticauda semifasciata*	2	1	19	37700	13.243	Basic PLA2
31	P20252	*Pseudechis australis*	3	2	19	10500	13.125	Basic PLA2
32	P29601	*Bungarus fasciatus*	2	2	12	11300	13.103	Acidic PLA2
**Three-finer toxins (3FTx): 33.70%**								
33	Q8JFX7	*Bungarus multicinctus*	2	2	17	105000	11.39	Muscarinic neurotoxin
34	Q9PW19	*Bungarus multicinctus*	2	2	17	105000	11.334	Cardiotoxin
35	Q9W7J5	*Pseudonaja textilis*	2	2	11	35500	11.269	Long-chain α-neurotoxin
36	A8HDK7	*Oxyuranus microlepidotus*	2	2	12	35500	10.308	Long-chain α-neurotoxin
37	A8HDK8	*Oxyuranus microlepidotus*	2	2	12	35500	10.267	Long-chain α-neurotoxin

38	C5ILC5	*Ophiophagus hannah*	1	1	11	29500	10.26	Long-chain α-neurotoxin
39	A7X4Q3	*Oxyuranus microlepidotus*	2	2	12	35500	10.237	Long-chain α-neurotoxin
40	A7X4R0	*Oxyuranus microlepidotus*	2	2	12	35500	10.198	Long-chain α-neurotoxin
41	A8HDK9	*Oxyuranus microlepidotus*	2	2	12	35500	10.183	Long-chain α-neurotoxin
42	F8J2E2	*Drysdalia coronoides*	1	1	11	29500	9.948	Long-chain α-neurotoxin
43	A6MFK5	*Demansia vestigiata*	2	2	12	35500	9.758	Long-chain α-neurotoxin
44	Q8AY49	*Bungarus candidus*	4	4	13	305000	9.758	Long-chain α-neurotoxin
45	A2CKF6	*Bungarus fasciatus*	2	2	15	78500	9.722	Long-chain α-neurotoxin
46	Q8AY51	*Bungarus candidus*	4	4	13	305000	9.713	Long-chain α-neurotoxin
47	A6MFK4	*Demansia vestigiata*	2	2	12	35500	9.705	Long-chain α-neurotoxin
48	Q9W729	*Bungarus multicinctus*	2	2	28	55200	9.657	Long-chain κ-neurotoxin
49	Q8AY56	*Bungarus candidus*	17	9	57	1980000	9.58	Long-chain κ-neurotoxin
50	Q9YGI8	*Bungarus multicinctus*	5	5	36	1320000	9.519	Short-chain α-neurotoxin
51	O12962	*Bungarus multicinctus*	5	1	38	38300	9.474	Long-chain κ-neurotoxin
52	A1IVR9	*Bungarus candidus*	1	1	13	29500	8.418	Long-chain α-neurotoxin

53	A1IVR7	*Bungarus candidus*	1	1	13	29500	8.35	Long-chain α-neurotoxin
54	A1IVR8	*Bungarus candidus*	1	1	13	29500	8.306	Long-chain α-neurotoxin
55	P01396	*Dendroaspis polylepis*	1	1	14	29500	8.043	Long-chain α-neurotoxin
56	C0HJD7	*Dendroaspis polylepis*	1	1	14	29500	8.002	Long-chain α-neurotoxin
57	P01397	*Dendroaspis polylepis*	1	1	14	29500	7.948	Long-chain α-neurotoxin
58	P25667	*Dendroaspis polylepis*	1	1	14	29500	7.939	Long-chain α-neurotoxin
**Kunitz-type serine protease inhibitor (Kunitz): 5.91%**								
59	B4ESA4	*Bungarus multicinctus*	2	1	20	236000	9.227	Kunitz
60	B4ESA3	*Bungarus multicinctus*	2	1	23	2770	9.158	Kunitz
61	Q8AY41	*Bungarus candidus*	2	1	23	2770	9.13	Kunitz
62	Q8AY43	*Bungarus candidus*	3	2	17	304000	9.077	Kunitz
63	Q8AY42	*Bungarus candidus*	3	2	17	304000	8.919	Kunitz

S2H. Venom composition of *B. fasciatus*

Sr.no.	Accession	Species	#Peptides	#Unique	Coverage (%)	Area	Avg. mass (KDa)	Toxin type
**Snake venom metalloproteinase (SVMP): 1.01%**								
1	A8QL49	*Bungarus multicinctus*	22	5	20	28600	68.99	SVMP
2	D5LMJ3	*Naja atra*	2	1	5	1290	68.25	SVMP
3	A8QL48	*Bungarus fasciatus*	26	9	33	102000	68.20	SVMP
4	Q10749	*Naja mossambica*	2	2	4	8820	68.18	SVMP
**Acetylcholinesterase (AChE): 2.14%**								
5	Q92035	*Bungarus fasciatus*	20	20	53	297000	68.07	Acetylcholinesterase
**5′-nucleotidase: 0.62%**								
6	B6EWW8	*Gloydius brevicaudus*	13	13	16	85500	64.43	5'-nucleotidase
**Phospholipase B (PLB): 0.08%**								
7	F8J2D3	*Drysdalia coronoides*	4	4	10	10700	64.10	Phospholipase B
**L-amino-acid oxidase (LAAO): 0.13%**								
8	A8QL52	*Bungarus fasciatus*	4	4	9	18700	58.76	L-amino-acid oxidase
**Venom nerve growth factor (NGF): 0.30%**								
9	P34128	*Bungarus multicinctus*	4	4	16	42200	27.51	Nerve growth factor
**Snake venom serine protease (SVSP): 0.07%**								
10	P00761	*Sus scrofa*	2	2	13	10200	24.41	Trypsin

**C-type lectins (CTL): 0.28%**								
11	Q90WI7	*Bungarus fasciatus*	8	8	57	39300	18.25	C-type lectin
**Phospholipase A2 (PLA2): 74.39%**								
12	Q75S48	*Bungarus candidus*	3	1	13	4270	16.86	Acidic PLA2
13	P00619	*Bungarus multicinctus*	1	1	15	671	16.22	Acidic PLA2
14	Q9DF33	*Ophiophagus hannah*	5	2	26	118000	15.90	Acidic PLA2
15	Q7T2Q4	*Bungarus flaviceps*	4	1	15	12600	15.88	Acidic PLA2
16	P30811	*Pseudonaja textilis*	1	1	13	27700	15.86	Acidic PLA2
17	P0C551	*Bungarus fasciatus*	14	11	75	412000	15.82	Acidic PLA2
18	Q90WA8	*Bungarus fasciatus*	78	14	79	1100000	15.77	Basic PLA2
19	Q7T3S7	*Echis carinatus*	1	1	14	30400	15.52	Acidic PLA2
20	Q6YNQ0	*Bungarus candidus*	1	1	16	671	15.09	Acidic PLA2
21	P14411	*Bungarus fasciatus*	25	18	72	1040000	14.84	Basic PLA2
22	P00629	*Bungarus fasciatus*	68	3	87	106000	14.75	Basic PLA2
23	P00628	*Bungarus fasciatus*	65	4	84	1970000	14.74	Basic PLA2
24	P00627	*Bungarus fasciatus*	77	12	84	4150000	14.70	Basic PLA2
25	P14615	*Bungarus fasciatus*	17	10	84	285000	13.43	Neutral PLA2
26	P00612	*Laticauda semifasciata*	3	1	19	6030	13.24	Basic PLA2
27	P20258	*Pseudechis porphyriacus*	3	1	20	11600	13.11	Basic PLA2

28	P29601	*Bungarus fasciatus*	20	16	74	1060000	13.10	Acidic PLA2
**Three-finer toxins (3FTx): 17.30%**								
29	Q2VBN2	*Ophiophagus hannah*	3	1	13	2290	9.82	Long-chain α-neurotoxin
30	A2CKF7	*Bungarus fasciatus*	11	10	48	700000	9.72	Long-chain α-neurotoxin
31	Q6IZ95	*Bungarus candidus*	11	10	48	700000	9.72	Long-chain α-neurotoxin
32	A2CKF6	*Bungarus fasciatus*	8	6	36	332000	9.72	Long-chain α-neurotoxin
33	P0C554	*Bungarus fasciatus*	5	5	23	178000	9.69	Short-chain α-neurotoxin
34	Q70WS8	*Bungarus multicinctus*	2	2	12	299	9.54	Long-chain α-neurotoxin
35	Q9YGI8	*Bungarus multicinctus*	3	3	12	188000	9.52	Short-chain α-neurotoxin
36	P0C552	*Bungarus fasciatus*	6	6	44	303000	8.70	Short-chain α-neurotoxin
**Kunitz-type serine protease inhibitor: 3.68%**								
37	B2KTG1	*Bungarus fasciatus*	10	10	48	511000	9.32	Kunitz
