# Supplementary material for: Beyond the ‘big four’: Venom profiling of the medically important yet neglected Indian snakes reveals disturbing antivenom deficiencies
Source: PLoS Negl Trop Dis. 2019 Dec 5;13(12):e0007899. doi: 10.1371/journal.pntd.0007899 (PMC6894822; doi:10.1371/journal.pntd.0007899)
Supplement: S3 Table — The following tables provide dose dependent effects of (A) Naja spp., (B) Bungarus spp., and (C) Echis subspecies venoms on extrinsic [Prothrombin time test (PT) and International Normalized Ratio (INR)] and intrinsic [Activated Partial Thromboplastin Time test (aPTT)] blood coagulation pathways. The delay in clotting time (or the time taken for the formation of the first fibrin strands), relative to the control sample in each test, is indicated by a color gradient from red to blue. *Blood clots immediately. (PDF) [file pntd.0007899.s009.pdf]

S3A. Table

Prothrombin time test (PT)

	N. naja (MH)	*N. kaouthia* (WB)	*N. kaouthia* (AR)
**Control**	14.5	14.5	14.5
**5 ug**	14	14.2	16.3
**10 ug**	15.9	14	15.3
**20 ug**	16.9	14.4	16.4
**40 ug**	18.2	14	15.8

International Normalized Ratio (INR)

	N. naja (MH)	*N. kaouthia* (WB)	*N. kaouthia* (AR)
**Control**	1	1	1
**5 ug**	0.96	0.97	1.14
**10 ug**	1.11	0.96	1.06
**20 ug**	1.19	0.99	1.15
**40 ug**	1.29	0.96	1.1

Activated Partial Thromboplastin Time test (aPTT)

	N. naja (MH)	*N. kaouthia* (WB)	*N. kaouthia* (AR)
**Control**	32	32	32
**5 ug**	56.7	30	32.7
**10 ug**	64.7	35.9	36.1
**20 ug**	77	39.5	40.9
**40 ug**	113.7	43.6	55.5

S3B. Table

Prothrombin time test (PT)

	B. caeruleus (PB)	*B. sindanus* (RJ)	*B. fasciatus* (WB)
**Control**	14.5	14.5	14.5
**5 ug**	13.7	15.1	14.1
**10 ug**	14.6	14.5	15.3
**20 ug**	13.6	14.5	14.5
**40 ug**	13.5	15.4	14.8

International Normalized Ratio (INR)

	B. caeruleus (PB)	*B. sindanus* (RJ)	*B. fasciatus* (WB)
**Control**	1	1	1
**5 ug**	0.93	1.04	0.96
**10 ug**	1	1	1.06
**20 ug**	0.93	1	1
**40 ug**	0.93	1.07	1.02

Activated Partial Thromboplastin Time test (aPTT)

	B. caeruleus (PB)	*B. sindanus* (RJ)	*B. fasciatus* (WB)
**Control**	32	32	32
**5 ug**	34.1	57.1	42.8
**10 ug**	34.2	63.2	55.4
**20 ug**	38.1	72.2	61.3
**40 ug**	45.9	78.5	68.2

S3C. Table

Prothrombin time test (PT)

	E. carinatus (MH)	*E. c. sochureki* (RJ)
Control	14.5	14.5
5 ug	0*	0*

*** Blood clots instantly**
