# Supplementary material for: Beyond the ‘big four’: Venom profiling of the medically important yet neglected Indian snakes reveals disturbing antivenom deficiencies
Source: PLoS Negl Trop Dis. 2019 Dec 5;13(12):e0007899. doi: 10.1371/journal.pntd.0007899 (PMC6894822; doi:10.1371/journal.pntd.0007899)
Supplement: S4 Table — A. Median lethal dose (LD50) of medically important snakes. This table provides LD50 values (in μg/mouse and mg/Kg) of various neglected snakes and their ‘big four’ counterparts. B. Median effective dose (ED50) of Premium serums antivenom. This table provides ED50 values and, estimated and marketed neutralizing potencies of the tested commercial antivenom (Premium Serums & Vaccines Pvt. Ltd.) against various species of medically important Indian snakes. For species, where the estimated neutralising potency meets the marketed potency of the commercial antivenom or that of its ‘big four’ counterpart [e.g., N. naja (0.60 mg/mL), and B. caeruleus and E. carinatus (0.45 mg/mL)], the cells are indicated in green. Venoms that are not neutralized by the antivenom in the mouse challenge model are indicated in light red. *antivenom failed to neutralize 5x and 3x LD50 venom doses. (PDF) [file pntd.0007899.s010.pdf]

S4A Table

	Dose of venom (ug)					Number of survivors					LD_{50} µg/mouse	LD_{50} mg/Kg
N. naja	9.6	12	15	18.7	23.4	5	5	3	0	0	14.57 (10.13 – 17.64)	0.73 (0.50 – 0.882)
N. kaouthia **West Bengal**	4.06	5.12	6.4	8	10	4	2	0	0	0	4.76 (4.38 – 5.37)	0.24 (0.18 – 0.28)
N. kaouthia **Arunachal Pradesh**	12.8	16	20	25	31.25	5	5	5	2	0	24.65 (22.80 – 26.65)	1.23 (1.14 – 1.33)
B. caeruleus	2.56	3.2	4	5	6.5	4	3	0	0	0	2.043 (0.66 – 6.28)	0.10 (0.03 – 0.31)
B. sindanus	0.25	0.4	0.6	1.024	1.63	4	2	1	0	0	0.37 (0.214 – 0.638)	0.02 (0.01 – 0.03)
B. fasciatus	12.8	16	20	25	31.25	5	5	1	1	0	22.36 (18.71 – 26.71)	1.12 (0.93 – 1.33)
E. carinatus	10.24	12.8	16	20	25	4	2	0	1	0	12.21 (6.88 – 15.01)	0.61 (0.34 – 0.75)
E. c. sochureki	25.6	32	40	50	62.5	5	3	2	0	0	35.01 (15.48 – 42.07)	1.76 (0.774 –2.10)

S4B Table

	Volume of antivenom injected in venom-antivenom mixture µL				ED50 µL	Potency of antivenom mg/mL	Marketed potency of antivenom mg/mL
N. naja	111.11	73.96	49.41	32.94	81.27 (67.30 – 98.13)	0.717 (0.59 – 0.87)	0.60
N. kaouthia **West Bengal**	166.67	111.11	73.96	49.41	122.04 (101.10 – 147.32)	0.156 (0.13 – 0.19)	–
N. kaouthia **Arunachal Pradesh**	166.67	111.11	73.96	49.41	ASV is not effective against the venom*	NA	–
B. caeruleus	32.94	21.97	14.64	9.76	26.17 (19.36 – 35.37)	0.312 (0.23 – 0.42)	0.45
B. sindanus	9.77	6.51	4.34	2.89	5.43 (4.34 – 6.51)	0.272 (0.23 – 0.34)	–
B. fasciatus	166.67	111.11	73.96	49.41	138.89 (111.11 – 166.67)	0.643 (0.54 – 0.80)	–
E. carinatus	166.67	111.11	73.96	49.41	92.54 (73.96 – 111.11)	0.527 (0.44 – 0.66)	0.45
E. c. sochureki	166.67	111.11	73.96	49.41	92.54 (73.96 – 111.11)	1.513 (1.26 – 1.89)	–
