# Supplementary material for: Beyond the ‘big four’: Venom profiling of the medically important yet neglected Indian snakes reveals disturbing antivenom deficiencies
Source: PLoS Negl Trop Dis. 2019 Dec 5;13(12):e0007899. doi: 10.1371/journal.pntd.0007899 (PMC6894822; doi:10.1371/journal.pntd.0007899)
Supplement: Attachment — Submitted filename: Response to Reviewers_PNTD comments.pdf [file pntd.0007899.s011.pdf]

Response to reviewer's comments

Reviewer 1

It appears that in their coagulation assays, the authors did not include calcium. This is critical as citrating plasma strips out the calcium, which must be added back in to restore physiological conditions. Otherwise this will dramatically skew the results. This has been shown previously to be a significant consideration with Echis venoms, which vary tremendously in their reliance on calcium and therefore the venoms will be differentially affected as the two species differ 4x in their relative reliance on calcium, and therefore the perceived potency significantly erroneous. Phospholipid dependency is another significant methodological shortcoming. As per: Differential procoagulant effects of saw-scaled viper (Serpentes: Viperidae: Echis) snake venoms on human plasma and the narrow taxonomic ranges of antivenom efficacies, as per: Rogalski A, Soerensen C, Op den Brouw B, Lister C, Dashevsky D, Arbuckle K, Gloria A, Zdenek CN, Casewell NR, Gutiérrez JM, Wüster W, Ali SA, Masci P, Rowley P, Frank N, Fry BG. Toxicol Lett. 2017 Oct 5;280:159-170

The conclusions in regards to the two Echis are skewed due to the methological issues, which will be remedied by including calcium and phospholipid in the protocol setup.

The results are well presented other than being erroneous in the coagulation analysing protocol.

We thank the reviewer for highlighting this important issue and for the appreciation of the importance of this study. We completely agree that the co-factors, such as calcium (Ca^{2+}) and phospholipids play a major role in the coagulation of blood. However, the commercial kits that were employed in our experiments (Agappe Cat #12602001), which are also used in human diagnostics, already contain the aforementioned cofactors, namely Ca^{2+} and phospholipids (rabbit brain cephalin). Please see the screenshot from the product description below. Hence, the results of our coagulation experiments are accurate and reliable.

Kit Components

Reagent/ Component	Product Code 12602001	Description
APTT Reagent 1	2 x 4 mL	Calcium chloride solution 0.020 M/L
APTT Reagent 2	2 x 4 mL	Rabbit brain cephalin<br>Ellagic acid activator<br>Buffer<br>Stabilizers and preservatives
Tri sodium citrate	1 x 10 mL	Tri sodium citrate (0.109 M/L) 3.2%

Also, the *Echis* procoagulant activity is well characterised as being driven by prothrombin activating P-III SVMP

We thank the reviewer for this suggestion. We have now added a line describing this with citation (line# 803).

Prior work that should be cited are:

We have now cited these suggested publications in our manuscript.

Line numbers – 754, 780, 803, 939.

Reviewer 2:

The issue raised by the article by Senji Laxme et al., entitled "Beyond the big four: Venom profiling of the medically important yet neglected Indian snakes reveals disturbing antivenom deficiencies" is crucial. The authors addressed the insufficiency of Indian antivenins manufactured to neutralize the venom from venomous snakes in India. Tested antivenoms did not cover some of the venoms from the 4 main species of Indian venomous snakes ('Big 4') coming from distant regions from the place of manufacture of the antivenoms. Moreover, antivenoms appeared to be ineffective against the venoms from other species of venomous snakes of India ('neglected species').

We are glad and thankful to the reviewer for considering some of the critical findings of our manuscript as important.

However, the authors subordinated the antivenom effectiveness to the composition of the venoms used for the manufacture. They deduced that poor efficacy of the antivenoms was based on venom composition variability. If this is probably one of the major reasons for the insufficiency of the antivenoms, there are other substantial causes, e.g. the manufacturing process of the antivenom and adherence to manufacturing standards. In this respect, contrary to what the authors claimed, the manufacture of antivenoms has evolved considerably since their invention in 1894 and we are currently using the fifth generation of antivenoms which are highly purified antibody fragments (at least when the manufacture is appropriate).

We completely agree with the reviewer that the antivenom manufacturing processes have greatly evolved over the past century. However, when we refer to antivenom manufacturing as a ‘century old strategy’ in our manuscript (line # 957), we are referring to the fact that we are still reliant on the use of animals for the antivenom production, instead of advanced recombinant technologies (line #956). In any case, following the reviewer’s suggestion, we have now removed the line referring to the antivenom production as a ‘timeworn strategy’ (line # 984). We also agree that it is not just the variability in the venom composition that can lead to inefficient antivenoms but also the implementation of appropriate antivenom manufacturing standards. We have therefore included a clarification in the line number 1001, which reads as below.

“In addition to geographic variability in venoms, the lack of adherence of Indian antivenom manufacturers to international production standards may also result in these undesirable outcomes.”

Regarding proteomic analysis of venom composition and antivenom neutralization tests, the methodology was reliable and relevant. However, for neutralization tests the use of antivenom dilutions leaded to challenge involving very small amounts of specific antibodies considering a) the low protein concentration of Indian antivenoms, and b) low proportion of specific antibodies relative to the amount of antivenom IgG, the majority of which being not directed against the venom).

We thank the reviewer for finding our methodology regarding proteomic analyses and antivenom neutralisation tests as reliable and relevant. We agree that the inefficiency of Indian

antivenoms is compounded by the dilution in these experiments. However, we did this to standardise the amount of antivenom immunoglobulins tested, and we followed WHO recommended preclinical neutralisation protocols (WHO, 2018), which require the administration of varying doses of the antivenom to calculate the 'effective dose'.

The results are clearly presented. The discussion is not relevant because only limited to composition of venoms as causes of insufficiency of antivenoms.

Under the section "Future directions for the production of highly efficacious paraspecific Indian antivenoms", from line number 954 to 1003, we have discussed the effects of other factors, including the failure of Indian antivenom manufacturers to meet the international antivenom production standards, exclusion of the 'neglected species' in the immunisation protocols, dose insufficiency of antivenoms, etc., as the underlying reasons that could result in such undesirable outcomes. Thus, we do not attribute the inefficiency of Indian antivenoms to the variability in the proteomic composition of venoms alone and therefore feel we have delivered a balanced assessment of this issue in the text.

However, the authors granted too much therapeutic efficacy to the antivenom. It is not a generic drug whose characteristics can be predicted by the choice of the venoms and methods of manufacture, nor even by the results of preclinical tests. The antivenom is a complex preparation the effectiveness of which stems from many parameters, starting with the venoms used to immunize horses, which the authors seek to evaluate, but also the production of antibodies by the horses, that nobody can anticipate, and the whole manufacturing process that leads to varying results regarding both efficiency and tolerance. The effectiveness of an antivenom is therefore not reproducible from a brand to another.

Thus, Authors' decision to test only one antivenom to preserve mice is an ethically virtuous decision but irrelevant on a methodological point of view because it is impossible to deduce the results of neutralization tests of antivenoms from different manufacturers on the basis of the result of a test performed with antivenom from a single manufacturer (Sánchez et al., Toxicon. 2015;106:97-107; Calvete et al. Toxicon. 2016;119:280-8).

Antivenom efficacy varies depending on the manufacturers and even batches from the same manufacturer. Horses' antibody production varies depending both on the immunogenicity of venom components and intrinsic individual immune response.

We thank the reviewer for these detailed comments. We completely agree with all the important points raised by the reviewer about the complications associated with the production of antivenoms. However, the ethical considerations of testing multiple batches of each Indian antivenom manufacturer (given the variation in immune responses of horses), and costs thereof, are beyond the scope and budget of this study. That said, we have now clarified that further preclinical and clinical research is necessary to effectively assess the efficiencies of the untested antivenoms in this study (line # 944), and we have cited the suggested references to emphasize this. In summary, we feel strongly that our in vivo experiments, while not as comprehensive as we would have liked, nevertheless provide an important contribution to the literature on the quality of antivenom available to treat Indian snakebite victims.

Horses produce on the one hand, different antibodies dependant to the structure of proteins (epitopes), and on the other hand, common antibodies to proteins belonging to the same family even if their mode of action is different (e.g. phospholipases A2 or 3-finger toxins: neurotoxins, cytotoxins, calciseptin, etc.). It has been demonstrated that distinct antigens (or antigens showing different symptoms) can induce a common antibody (Rousselet et al. Eur J Biochem. 1984;140:31-7) and, conversely, single antigen can result in the production of antibodies recognizing many variants of the antigen, including from distinct species (Tremeau et al. FEBS Lett. 1986;208(2):236-40). If the authors are right to write "presence of a wide range of toxin types, capable of exerting variable clinical pathologies following snakebite", which involves a vast range of antibodies, it is also true that many toxins belonging to the same family of proteins (e.g. 3-finger toxins) can be neutralized by a limited number of antibodies. The authors did not seek antigenic kinship between the components of venoms from different species, which would have made it possible to define, perhaps, cross reactions among the antibodies... Amino acids that modify the functioning of the protein (eg substitution of the 49th residue of phospholipase A2), are they included in the epitope that directs the making of the antibody?

We fully agree with the reasoning above. However, we sought to determine whether commercially available antivenoms, which are produced only against the ‘big four’ snakes, are capable of effectively cross neutralising the bites of the neglected snake species that contain venom protein groups that are also present in venoms of the ‘big four’. As demonstrated, although the tested antivenoms show cross reactivity in certain *in vitro* experiments (ELISA and Western Blotting), at least one of the antivenoms tested fails to neutralise bites from the

neglected species in the mouse model (ED_{50} experiment). Therefore, although immunological cross reactivity is interesting and informative from a scientific perspective, we found that it is imprudent to use these results to predict the preclinical efficacy of an antivenom to venoms of the 'neglected many' species under investigation.

In addition, preclinical tests use experimental models (mice) that are not predictable of outcomes in humans. An effective antivenom in mice may not be in humans and vice versa. As a result, the definitive sanction is provided only by clinical trials.

We fully agree that clinical trials are vital. However, given the complexity of the antivenom manufacture as pointed out by the reviewer, performing clinical research on every batch of antivenom is clinically and logistically impossible. Instead, once a clinical trial has proven efficacy, the WHO recommended preclinical assays used by both Indian antivenom manufacturers and researchers around the world (1-3) are very useful quality control tools to monitor (i) batch-batch variation of a product, and (ii) compare efficacies of multiple products. Moreover, despite being marketed for several decades, the Indian antivenoms have never undergone scientifically robust clinical trials. Therefore, we believe that preclinical research can be the first step to highlight the pitfalls of the currently marketed antivenoms, and to pave way for clinical research.

Venom variability has been shown to be almost as important at the specific, geographic, and individual levels (Chippaux et al., Toxicon, 1991: 29: 1279-303), making the manufacture of antivenoms complex and random the effectiveness during envenomation. The response of an antivenom to various venoms from individuals of the same species but from distinct geographical origins, or from distinct individuals from the same region has been studied in detail (Roodt et al. Toxicon. 2011;(7-8):1073-80).

We fully agree with this, and we have now supported similar statements in the manuscript by including the aforementioned citations (line 865, 1001).

Moreover, the influence of the diet on the composition of the venom remains a hypothesis, and one could just as well say that the composition of the venom is the result of the chance of genetic mutations and gene captures (Casewell et al. Nat Commun. 2012;3:1066), and that it influences the

diet, not the other way around. The section on venom variability according to ecology is certainly an interesting hypothesis but not very relevant considering the toxicity of venom since it is not transferable from one animal model to another - and therefore to the human.

We thank the reviewer for their considered comments on the influence of diet on venom. However, mutations in venom coding genes can certainly accommodate shifts in feeding ecologies (Sunagar & Moran, 2015) but a shift in feeding ecology is unlikely to be triggered by genomic mutations. We also feel that feeding ecology of the venomous animal often plays an extremely important role in the medical relevance of the species against humans. For example, in this study, we clearly see that *E. c. sochureki* and *B. fasciatus* – scorpion and snake specialist, respectively, show decreased toxicities against mammals. In contrast, the venoms of mammalian specialists like the *N. naja* and *E. carinatus carinatus* secrete medically important venom cocktails. Therefore, after very careful consideration, we elect to retain our position on this issue.

The poor neutralization of venoms from neglected species by Indian antivenoms deserves further investigation and, as the authors emphasized, to seek a solution to remedy it. It appeared that the cross-reactions between the antibodies of some of the antivenoms and the venom of species not used for the immunization of horses were restricted. The ELISA tests constitute a first evaluation of the recognition of these venoms by the antibodies.

However, only the neutralization tests make it possible to say a) if the antibodies show partial efficacy and what is the titer, b) whether they are toxic antigens or not (at least for the experimental model, i.e. the mouse, and (c) what would be a priori the dosage to consider. It is nevertheless clear that these tests are only indications that make it possible to envisage the inclusion of more or less significant amount of venoms of the neglected species in the cocktail of immunization of horses.

The recommendation to Indian manufacturers of antivenoms should be to diversify the sources of their venoms and add some neglected species.

We fully agree with this point and we have indeed highlighted the same in line #981

A wise choice would be to add not the most common species but those that make it possible to obtain the widest range of antibodies in order to cover -by cross-reactions- the largest number of

species. However, one does not exclude the other, since the final result in humans may not be anticipated.

The research strategy suggested by the reviewer is very sound and an area we feel our study makes a useful contribution. Unfortunately, given the lack of such studies in the Indian subcontinent, species that can elicit the widest range of antibodies are yet to be identified. We are hopeful that, by publishing the work of this study, we will help pave the way for more, urgently-needed research as identified by the reviewer.

Other Changes

- Changed the colour of the India map in Figure 1.
- A ‘Striking Image’ has been uploaded for the manuscript

References

Casewell, N. R., Cook, D. A., Wagstaff, S. C., Nasidi, A., Durfa, N., Wuster, W., & Harrison, R. A. (2010). Pre-clinical assays predict pan-African Echis viper efficacy for a species-specific antivenom. *PLoS Negl Trop Dis, 4*(10), e851. doi:10.1371/journal.pntd.0000851

Gutierrez, J. M. (2018). Preclinical assessment of the neutralizing efficacy of snake antivenoms in Latin America and the Caribbean: A review. *Toxicon, 146*, 138-150. doi:10.1016/j.toxicon.2018.02.053

Harrison, R. A., Oluoch, G. O., Ainsworth, S., Alsolaiss, J., Bolton, F., Arias, A. S., . . . Casewell, N. R. (2017). Preclinical antivenom-efficacy testing reveals potentially disturbing deficiencies of snakebite treatment capability in East Africa. *PLoS Negl Trop Dis, 11*(10), e0005969. doi:10.1371/journal.pntd.0005969

Sunagar, K., & Moran, Y. (2015). The Rise and Fall of an Evolutionary Innovation: Contrasting Strategies of Venom Evolution in Ancient and Young Animals. *PLOS Genetics, 11*(10), e1005596. doi:10.1371/journal.pgen.1005596

WHO. (2018). World Health Organisation guidelines for the production, control and regulation of snake antivenom immunoglobulins, 2018. In.
